# Incidence of Characteristic Findings during Veterinary Carcass Inspections 2010–2019 in the Czech Republic and the Relation to the Level of Health and Welfare of Individual Classes of Cattle

**DOI:** 10.3390/ani11020537

**Published:** 2021-02-19

**Authors:** Michal Kaluza, Vladimir Vecerek, Eva Voslarova, Zbynek Semerad, Annamaria Passantino

**Affiliations:** 1 Department of Animal Protection and Welfare and Veterinary Public Health, Faculty of Veterinary Hygiene and Ecology, University of Veterinary and Pharmaceutical Sciences Brno, 612 42 Brno, Czech Republic; vecerekv@vfu.cz (V.V.); voslarovae@vfu.cz (E.V.); 2 Central Veterinary Administration of the State Veterinary Administration, 120 00 Prague, Czech Republic; z.semerad@svscr.cz; 3 Department of Veterinary Sciences, University of Messina, Polo Universitario Annunziata, 981 68 Messina, Italy; passanna@unime.it

**Keywords:** slaughter, veterinary inspection, pathological findings, health, welfare

## Abstract

**Simple Summary:**

The data from a veterinary inspection is a valuable tool in monitoring the health and welfare of slaughtered animals and can provide information relevant to the assessment of their living conditions. In our study, the results of veterinary inspections of cattle slaughtered in the Czech Republic in the period 2010–2019 were analyzed. In terms of localization, the most frequent findings in cattle were in the liver and pancreas (26.36%), in the lungs (25.44%) and in the urinary tract (25.19%). Among adult cattle, according to these pathological findings, cows were numerically the most affected class. In contrast, bulls had very good levels of health. In calves, the most frequent were findings in the lungs, unclassified changes and overall changes. The results showed the different health issues and the different states of health in the individual classes of cattle, which demand a different approach to the treatment and prevention of the most frequent diseases. The spectrum of characteristic findings is also a reflection of the level of welfare. Deteriorated living conditions should be mentioned especially in cows but also in calves which often suffered from emaciation or stunted growth.

**Abstract:**

Pathological findings in individual classes of cattle were assessed from the viewpoint of their localization and category. The objective of the study was to evaluate whether there are differences in the range and number of findings made between the individual classes of cattle. The results of veterinary inspections on 2,514,666 head of cattle slaughtered in the Czech Republic in the period 2010–2019 were used for the assessment. In terms of localization, the most frequent findings in cows were in the liver and pancreas (46.13%), the urinary tract (40.76%) and the lungs (36.23%). These findings also predominated in heifers and bulls, though they were recorded at lower frequencies (*p* < 0.01) than in cows. The most frequent pathological changes in heifers and bulls were chronic findings in the lungs (16.09% and 12.27%, respectively). The range of findings in calves differed significantly from other classes of cattle, primarily as the result of respiratory and diarrheal syndrome being the most frequent diseases in calves. Calves were the class of cattle most frequently diagnosed with findings in the lungs (44.89%), as well as other unclassified changes (24.43%) and overall changes (21.55%), which point to a systemic disorder of the organism. The results of this study confirmed the differing states of health in the individual classes of cattle and the differing health issues to which treatment and the prevention of the most frequently occurring infectious and non-infectious diseases must be adapted. Cattle welfare is affected not only by the level of health but also by the herd management and economics. This is confirmed by the range of findings, and the deterioration of living conditions especially in cows, likely because of great intensity of farming, but also in calves which suffered from emaciation or stunted growth.

## 1. Introduction

Veterinary inspections of animals and animal products is one of the most widespread and longest-functioning surveillance systems [1]. The data from veterinary inspection is a valuable tool in monitoring the health and welfare of slaughtered animals, and can provide information relevant to the assessment of their living conditions [2,3]. Serious violations of the level of welfare may lead to changes in the organism, which are then made evident by findings at the slaughterhouse [4,5]. Assuring welfare is not merely important from the viewpoint of animal protection and the ethics of our treatment of animals, but also has an impact on the attainment of meat of high quality from slaughter animals. Inappropriate living conditions lead to chronic stress in animals and the possible development of medical problems. Assessing welfare is not merely a matter of the ethics of a farmer’s approach to animals, the elimination of welfare problems may lead to improved production on farms [6].

Livestock welfare has become a matter of public concern and consequently, animal production has become subject to a more rigorous scrutiny due to EU legislation [7]. According to Velarde and Dalmau [8], people are convinced of the importance of good living conditions for farm animals and are willing to consider this in their buying strategies with respect to animal products.

A carcass inspection not only provides an awareness of the quality and safety of animal products, but also gives a comprehensive overview of the slaughtered animal’s health [9]. Klink et al. [10] emphasized the huge amount of information that can be obtained at the slaughterhouse about the state of health of slaughtered animals and about findings that may be associated with the level of welfare of reared animals. Findings made at the slaughterhouse can be linked with the diagnostics of the herd and can be used to gain comprehensive information about medical problems on individual farms. Feedback from slaughterhouses is of indisputable importance to successful herd management [11].

The frequency and range of pathological changes in cattle are often different in different countries [9]. Ceccarelli et al. [12] performed retrospective analysis of the results of inspections on slaughter animals from the period 2010–2016. They analyzed the reasons for the condemnation of carcasses and organs at selected slaughterhouses in central Italy. The results of their study show that the most frequent findings leading to condemnation were lesions in the lungs (64.86% of cases), in the liver (31.20% of cases) and in the stomach (11.63% of cases). Cerchiaro et al. [13] determined the reasons for culling beef bulls in Italy. They found the main reasons for culling to be disorders of the musculoskeletal system (42%) and respiratory system (24.3%). Tabaran et al. [14] assessed findings at Romanian slaughterhouses in the years 2016–2017. The most frequent were findings of parasitic origin in the lungs. Another frequent finding in the lungs was pneumonia (23.2%). The liver was also an organ with the most frequent parasitic lesions. Dupuy et al. [15] analyzed findings at slaughterhouses in France during 2005–2010. Their results showed that 68% of the cattle with at least one condemned portion were related to condemnation of the liver. Less frequent findings of the reason for condemnation were made in the kidneys (19.23%) and lungs (18.1%). Januskeviciene et al. [16], who studied the incidence of pathological findings in cattle in Lithuania in 2000–2009, reached similar conclusions. The authors noted that most findings were made in the liver (25.09%) and the respiratory tract (20.44%). Less common were findings in the digestive system (10.49%), the kidneys (9.6%) and the heart (8.41%). Blanco-Penedo et al. [17] compared organic farms with intensive and conventional beef farms in Spain in 2007. On the basis of their findings, they concluded that the level of health between these systems did not differ significantly. Results from slaughterhouses showed that a total of 26% of all animals had at least one pathological finding. Lesions were found most often in the lungs (25.71%), the liver (14.09%), the kidneys (11.31%) and the digestive tract (5.75%), regardless of the farming system. The health of the animals is influenced more by the management of the specific farm and the approach taken by the farmer than by the husbandry systems used [18]. Other studies have been contradictory, indicating that mastitis, laminitis and lesions on the limbs are a less serious problem on organic farms than they are in conventional farming [19].

Some studies have focused on certain groups of findings that may point to problems with the living conditions of the animals on farms. Bethancourt-Garcia et al. [20] studied the occurrence of bruises in bulls and cows. They found a far lower occurrence of bruising in bulls (17.2%) than in cows (38.6%). Brscic et al. [21] studied the effect of housing systems on the health and welfare of bulls in Italy. They found bulls housed on slatted flooring to be culled earlier than bulls housed on deep litter as a result of the higher incidence of bursitis, swellings and alopecia. The results of their study confirmed that pathologies of the musculoskeletal system were a more frequent cause of culling on farms with slatted floors (87.8% of cases) than on farms using a deep litter system (75% of cases).

Assumedly, the different classes of cattle (calves, heifers, cows and bulls) slaughtered in the slaughterhouse will also differ in the incidence of pathological findings. This has, to the best of the authors’ knowledge, not been presented in the literature before.

The aim of this study was to assess the characteristic findings made during post-mortem veterinary examination of cattle slaughtered at slaughterhouses in the Czech Republic in the period from 2010 to 2019 according to their localization and category of lesions in individual classes of cattle. Furthermore, the study was to evaluate whether there are differences in the range and number of findings made between the individual classes of cattle. Findings that may be associated with violation of the welfare of slaughtered animals were also assessed.

Cattle farming in the Czech Republic has seen a significant declining trend in the last 30 years (from 3,506,222 cattle in 1990 to 1,404,117 in 2020). In 2019, about 17% of farmed cattle were kept for meat production. Average weight of bovine animals slaughtered in the Czech Republic is 680 kg for bulls, 540 kg for cows and 475 kg for heifers. Bulls and heifers in fattening systems are slaughtered under the age of two years (12–24 months depending on the breed). Young cattle (8–12 months) are slaughtered rarely [22,23]. Male dairy calves are often sold for fattening abroad. Females are used for future dairy production. Most calves slaughtered in the Czech slaughterhouses originate from the dairy industry. Small farmers represent a majority of the Czech cattle farming sector [24]. Beef cattle are reared mainly in extensive farming systems, in other words, kept on pastures for most of the year. Most Czech dairy cows are kept in barns with a capacity of 255 cows and above (the average herd size is 314 cows per farm) [22].

## 2. Materials and Methods

Data for analysis was obtained from the information system of the State Veterinary Administration of the Czech Republic, which contains information on the number of slaughtered animals and the results of veterinary inspections recorded by official veterinary inspectors of the Regional Veterinary Administrations of the State Veterinary Administration operating at slaughterhouses in the Czech Republic. Data on the results of veterinary inspections on cattle slaughtered at all slaughterhouses in the Czech Republic in the period from 2010 to 2019 was used for the evaluation. A total of 2,514,666 cattle (1,136,754 cows, 257,912 heifers, 1,015,541 bulls and 104,459 calves) transported from the Czech cattle farms were slaughtered at 226 Czech slaughterhouses in the monitored period (41.62% of animals were slaughtered in the six largest slaughterhouses each having capacities of more than 10,000 slaughtered bovine animals per year). Only animals considered fit to be transported to the slaughterhouse (according to the Council Regulation (EC) No. 1/2005 [25] and slaughtered for human consumption were included in the analysis. Pathological changes were evaluated separately for individual cattle classes, namely, cows, heifers, bulls and calves, slaughtered at the slaughterhouses. The individual classes of cattle were defined as follows: calves (up to 6 months of age), heifers (females from 6 months of age to the first calving), cows (females after the first calving) and bulls (males over 6 months of age).

The spectrum and frequency of pathological findings were assessed for each class of cattle. Pathological changes were classified according to localization, in other words, the place of occurrence of findings. Groups of findings, according to their localization in cows, heifers, bulls and calves, were then specified according to the category of lesions.

Pathological findings on the organs were categorized either as acute, chronic or parasitic. Pathological changes in the body and limbs were categorized either as acute, chronic or traumatic.

Pathological changes caused by an inflammatory process lasting a short period before slaughter were classified among acute findings. These findings included marked hyperemia, the presence of hemorrhages, swelling, increased organ size, the presence of catarrhal, hemorrhagic, purulent or fibrinous exudates, et cetera.

Pathological changes caused by an inflammatory process lasting for a prolonged period before slaughter were classed as chronic findings. These findings included changes to the original structure of tissue parenchyma involving penetration of connective tissue, the formation of connective tissue scarring and the appearance of adhesions. The chronic findings were also indicated by reduction to the size of organs and their stiffness, change to the structure of the mucous and serous membrane surfaces in the sense of their roughening, the presence of post-inflammatory cavities, and the presence of cysts or calcifying abscesses, et cetera.

Changes that pointed to the invasion and migration of parasites and pathological processes caused by parasites in the host organism were classed as parasitic findings. Primarily, this involved cysticercosis and the presence of cysticerci at predilection sites—the heart, the esophagus, the diaphragm, the sublingual area and the masticatory muscles. *Sarcocystis* and the occurrence of nematodes in the respiratory tract were other parasites monitored during veterinary inspection.

Traumatic changes were not included in acute or chronic changes but were recorded separately in order to determine the extent of the traumatic lesions as a part of animal welfare inspection. Findings of traumatic origin were considered to be changes (acute or chronic) represented by open wounds at various stages of healing, hematomas in the subcutaneous tissue and muscles, contusions, dislocations, fractures and other changes that could have been caused directly by husbandry technology, improper human handling or other animals on the farm, in transport or lairage. Post-mortem changes resulting from stunning were not included.

Overall changes included findings involving the whole organism of the animal, such as stunted growth, emaciation, ascites, occurrence of abscesses on many organs, tuberculous changes and others.

Other changes included other severe pathological changes in muscles and organs of acute, chronic or parasitic character (such as findings on pleura, peritoneum, diaphragm, hernia, etc.).

Unclassified changes included pathological findings which were not related to a specific organ or tissue, namely, unspecified morphological formations, tumors, color changes and other changes that could not be included in any other category.

The most frequent types of findings were determined in the individual classes of cattle and compared between classes. The results were evaluated statistically using the program Unistat 6.5 for Excel (Unistat Ltd., London, UK, 2013). For the purposes of statistical comparison of the frequency of findings in individual animal classes, a chi-square test was used to assess statistical significance in a 2 × 2 contingency table. Yates’ correction was used on frequencies exceeding 5, while Fisher’s exact test was used at frequencies less than 5. Spearman’s rank test was used to assess the trend between individual years of the monitored period in the number of selected findings (limbs). The result of testing was determination of rank correlation coefficients (Spearman’s rank correlation coefficient = rSp) used to assess a positive or negative trend in the numbers of findings.

## 3. Results

During the period 2010–2019, 1,136,754 cows, 257,912 heifers, 1,015,541 bulls and 104,459 calves were slaughtered at slaughterhouses in the Czech Republic. A comparison of the number of pathological findings according to their localization in individual classes of cattle is given in Table 1.

The results show that, from the viewpoint of the localization of findings, the most frequent findings in cattle were in the liver and pancreas (26.36% of all animals), in the lungs (25.44% of all animals) and in the urinary tract (25.19% of all animals). The most frequent findings in cows were in the liver and pancreas (46.13%), the urinary tract (40.76%) and the lungs (36.23%). Findings in the liver, lungs and urinary tract also predominated among heifers and bulls, though they were less frequent than in cows. The most frequent pathological changes in heifers were in the lungs (17.61%), less frequent were findings in the urinary tract (16.25%) and liver (14.79%). The most frequent pathological changes in bulls were findings in the lungs (13.34%), in the urinary tract (10.87%) and in the liver (8.12%). The range of findings in calves differed markedly from the other classes. The findings most frequently diagnosed were in the lungs (44.89%). In contrast to other classes of cattle, unclassified changes (24.43%) and overall changes (21.55%), including emaciation, stunted growth, abscesses, ascites and tuberculous changes, were often recorded in calves. Differences in the frequency of individual findings were statistically highly significant (*p* < 0.01) between classes of cattle with the exception of findings in the central nervous system (CNS). Statistically significant (*p* = 0.02) differences were found between cows and heifers in the case of findings in the CNS while no statistically significant differences (*p* > 0.05) were found in findings in the CNS between cows and calves and between heifers and calves.

A comparison of the frequency of pathological findings in the liver according to the category of lesions in individual cattle classes is given in Table 2. In cows, the most frequent pathological findings in the liver were chronic findings (38.66%). Chronic findings in the liver also predominated in the other classes of cattle, though the recorded frequencies were not as high as in cows. The fewest chronic findings in the liver were seen in bulls (7.12%). Differences in the frequencies of the individual types of findings in the liver were statistically highly significant (*p* < 0.01) with the exception of comparison between parasitic findings and icterus in calves where no significant (*p* > 0.05) difference was found. Classes of cattle also differed (*p* < 0.01) from one another in the frequency of individual types of findings in the liver. Exceptions to this were acute findings in the liver, for which no significant differences (*p* > 0.05) were found between cows and calves. No statistically significant differences between heifers and calves were demonstrated when chronic findings and findings of icterus were compared (*p* > 0.05).

A comparison of the occurrence of pathological findings in the lungs according to the category of lesions in cattle is given in Table 3. The results show that chronic findings predominated in all classes of cattle. The most affected were calves (35.63%), in which acute changes in the lungs (9.22%) were also detected most frequently of all the cattle classes. Frequent chronic changes in the lungs were also recorded in cows (29.92%). The lowest number of chronic findings in the lungs was in bulls (12.27%), in which the fewest acute findings were also recorded (1.05%). Parasitic findings in the lungs were detected to only a small extent in all classes of cattle. Differences in the frequencies of acute, chronic and parasitic findings in the lungs were statistically highly significant (*p* < 0.01). Classes of cattle also differed (*p* < 0.05) from one another in the frequency of individual types of findings in the lungs except that no statistically significant (*p* > 0.05) differences were found between heifers and bulls in parasitic findings.

A comparison of the occurrence of pathological findings in the kidneys between individual classes of cattle is shown in Table 4. Chronic findings predominated in the kidneys in all classes of cattle. The largest number of chronic findings in the kidneys was seen in cows (36.76%). The lowest number of chronic findings was recorded in bulls (10.42%). Differences in the frequency of acute, chronic and parasitic findings in the kidneys were statistically highly significant (*p* < 0.01). Individual cattle classes also differed (*p* < 0.05) from one another in the frequency of individual types of kidney findings with the exception of heifers and bulls in the case of parasitic findings.

A comparison of the incidence of pathological findings in the limbs in individual classes of cattle is given in Table 5. Chronic findings predominated in all classes of cattle. The highest frequency of chronic findings in the limbs was recorded in cows (7.18%). Calves were another group affected by chronic changes in the limbs (4.16%). The lowest number of all pathological findings in the limbs was recorded in bulls. Differences in the frequency of acute, chronic and traumatic findings in the limbs were statistically highly significant (*p* < 0.01). All classes of cattle also differed (*p* < 0.01) from one another in the frequency of individual types of findings in the limbs.

The trend in the numbers of findings in limbs was also monitored in the individual classes of cattle. A negative correlation was found in cows (rSp = −0.806, *p* < 0.01), calves (rSp = −0.685, *p* = 0.03) and heifers (rSp = −0.673, *p* = 0.03). Bulls were the only class without a statistically confirmed declining trend of numbers of findings in the limbs.

Among the findings associated with overall changes on the body, emaciation, stunted growth and abscesses were studied. A comparison of the occurrence of these changes between individual classes of cattle is given in Table 6. Emaciation was found most frequently in calves (17.04% of animals). Other classes of cattle did not show signs of emaciation at such high frequencies. Stunted growth was also noted in connection with emaciation, and was likewise detected most frequently in calves (3.49%). Abscesses were only recorded at low frequencies. They were detected most often in cows (1.85%), and least often in heifers (0.70%). Differences in the frequency of findings of abscesses, emaciation and stunted growth were statistically highly significant (*p* < 0.01). Individual cattle classes also differed (*p* < 0.01) from one another in the frequency of individual types of findings categorized as overall changes with the exception of heifers and bulls in case of abscesses.

## 4. Discussion

Each farmer should devote attention to the possible interaction between animal health and the need for assuring good living conditions [6]. An inadequate level of welfare may weaken the immune system of the individual and thereby increase the risk of occurrence of diseases, which is then manifested in the presence of characteristic findings during veterinary inspections. The results of the studies published by Lis [26,27,28] and other authors point to the fact that pathological findings occur in a large number of the organs of the carcass. The results of our study show that differences exist between the individual classes of cattle in the spectrum and frequency of these findings, which points to different health issues and a different level of health in the individual classes of cattle. Overall, however, the most common in all classes of cattle were findings in the liver and pancreas (26.36% of all animals), the lungs (25.44% of all animals) and the urinary tract (25.19% of all animals). Similar conclusions were also reached by Januskeviciene et al. [16], who detected the largest number of findings in the liver (25.09%) and respiratory tract (20.44%). Findings in the kidneys (9.6%) in their study were not as frequent as in our study. Findings in the liver and lungs were also reported by Ceccarelli et al. [12] and Dupuy et al. [15] as the most frequent reasons for condemnation.

Findings in the liver and pancreas in all classes of cattle were predominately chronic. The group most affected by the occurrence of chronic findings in the liver and pancreas were cows (Table 2). Such high frequencies were not recorded in other classes of cattle, which points to the high intensity of exploitation of cows on farms which has an impact on the organisms’ metabolic centers. Dairy cows, in particular, are exposed to considerable metabolic burden. A negative energy balance occurs in high-yielding dairy cows within two weeks of calving. If they are fed improper rations (overfeeding) during the dry period, they are under a considerable risk of lipomobilization syndrome and hepatic steatosis after calving. Gonzalez et al. [29], who studied the connection between the blood indicators of lipomobilization and hepatic function in high-yielding dairy cows found that 30% of cows suffered from hepatic steatosis during early lactation. Liver damage and impaired hepatic function were confirmed in the results of laboratory tests by high levels of the activity of aspartate transaminase (AST) and low levels of glucose, total protein and urea. Raoofi et al. [30], who assessed fatty liver in Holstein dairy cattle at slaughterhouses in Tehran, also pointed out the development of hepatic steatosis after calving. Postparturient cows had the highest concentration of fat in the liver of all studied classes (65% of animals had hepatic steatosis). Only around 20% of pregnant cows, cows in the dry period and cows that were not pregnant had hepatic steatosis. High milk production is thus reflected in typical findings in the carcass, particularly in the liver, though also in the kidney, and the results of our study support this.

Findings in the kidneys were most frequently chronic in all classes of cattle. Cows were most affected, with a high frequency of chronic findings recorded (Table 4), which is connected with the substantial metabolic burden on dairy cows in particular, as mentioned above. The fewest chronic findings in the kidneys were recorded in bulls (10.42%). Findings in the kidneys are often linked with the intensity of fattening, when the formation of uroliths and bladder infections may occur in young animals during fattening to the age of two years, as reported by Herenda et al. [31]. The frequency of incidence of chronic findings in the kidneys, both in bulls and in other classes of cattle in our study, supports their claim.

Findings in the lungs were most frequently detected in calves (Table 1) and chronic findings predominated in this class of cattle (35.63% of calves). Chronic findings also predominated in the lungs in other classes (Table 3). In calves, however, acute changes in the lungs were also detected more frequently than in any other class (9.22% of calves). The high frequency of pathological findings in the lungs is the result of the occurrence of respiratory syndrome which, along with diarrheal syndrome, is one of the most frequent diseases in calves. Secondary pneumonia occurs in calves as a sequel to, or simultaneously with infectious diarrhea [32]. Respiratory diseases in calves generally appear in group housing, to which the calves are relocated from individual boxes. The same scenario may occur when calves are housed under a common roof, in other words, in calf sheds. Bojkovski et al. [33] reported that the crowded spaces with little ventilation in which calves are housed are, along with weakened immunity, substantial factors in the development of respiratory symptoms.

Findings in the lungs in calves are more frequent in intensive farming than in extensive farming. Taylor et al. [34] mentioned the purchase of calves from herds with a different epidemiological situation as one of the risk factors. Some other risk factors they mentioned include low weight, compromised immunity and the transport of animals, which can lead to an outbreak of symptoms of respiratory disease following arrival at the herd or collection center or after transport to the slaughterhouse. If treatment is not commenced in time, the calves do not thrive and become emaciated, which may be reason for their being discarded from the herd and taken to the slaughterhouse, where both changes in the lungs and emaciation or stunted growth are then determined. Diseases of the gastrointestinal tract are also a big problem in calves. The results of this study show, however, that in comparison with respiratory diseases they are not the primary reason for culling calves. Findings in the stomach and intestines in calves were recorded in only low numbers (1.26%; 2.30%). Clean and dry bedding is of great importance in preventing these diseases [35].

Frequent chronic changes in the lungs were also recorded in cows and heifers (Table 3). This indicates a problem with the treatment of respiratory disease which, if not rigorous, may lead to the chronic expression of the disease. Such disease is then manifested in characteristic findings in the lungs. Another reason for the occurrence of respiratory diseases in older classes of cattle may be unsuitable animal hygiene conditions in stalls, which are substantial predisposing factors for the development of symptoms of respiratory diseases.

The fewest chronic findings in the lungs were detected in bulls (12.27%), as were the fewest acute findings (1.05%), which confirms the fact that housing on pastures, which predominates in the extensive farming of fattening bulls in the Czech Republic [24], is appropriate from the viewpoint of prevention of respiratory diseases. The risk of respiratory diseases increases during housing on deep litter, which is used primarily in the winter, as was also confirmed by Brscic et al. [21]. While housing on deep litter is far more favorable than housing on slatted floors from the viewpoint of prevention of lameness, it is essential to maintain the appropriate level of hygiene and cleanliness to avoid the risk of respiratory diseases. The results of their study focusing on the causes for the culling of bulls indicate that respiratory symptoms were a more frequent reason for culling on deep litter (25% of cases) than in herds housed on slatted floors (2.4% of cases). High atmospheric humidity or high dustiness levels may also be a cause of pulmonary emphysema [15]. Respiratory diseases in bulls have also been studied by Cerchiaro et al. [13], who investigated the reasons for culling in herds of beef cattle in Italy. The authors state that, along with musculoskeletal problems (42% of cases), respiratory diseases (24.3% of cases) were the main reason for the culling of fattening bulls on 29 farms. These results were corroborated by our study, where the most frequent findings in bulls according to localization were lesions in the lungs (14.26%). It can, then, be assumed that if bulls were culled at lower weight than their standard slaughter weight, the reason for culling may have been respiratory diseases. It must be stated here that bulls are in very good health from the point of view of the types and frequency of other findings in comparison with other classes of cattle.

Findings on the limbs may be associated with the possible welfare problems. Inadequate flooring [36] or housing system [37] may result in injuries. Any injuries causing acute or chronic pain negatively affect the welfare of animals [8]. Furthermore, traumatic findings not only point to an impaired welfare of animals but also present significant economic losses for farmers [38]. Such findings reduce the quality and thus the value of the carcass [39]. According to Weary and Taszkun [37], lesions on cattle are most likely to occur near areas of the body where there is some protrusion, such as at the joints, including carpal, fetlock, tarsal and hip. Their findings are in agreement with our results as traumatic findings were more frequently recorded on the limbs than on the body in all classes of cattle.

Uneven load on the limbs also poses the risk of traumatic changes such as pressure sores and bruises, and not merely on the limbs. The risk of occurrence of lesions on the limbs also depends on the quality of bedding. High humidity, muddy areas and insufficient bedding may lead to hoof diseases in cattle [40]. According to Bergsten [41], the risk of hoof diseases and the risk of lameness are related to the intensity of milk production and the farm management. The occurrence of limb diseases is greatly affected not only by genetics, but also nutrition, animal hygiene and herd management [41]. Jewell et al. [42] found knee (carpus) lesions to be more frequent in tiestalls (17% of animals) than in freestalls (14% of animals). In general, older cows have a higher occurrence of lesions on the knees and neck.

Musculoskeletal problems were also studied by Brscic et al. [21] who found that they were a less frequent reason for culling in deep litter systems (75.0% of cases) than in slatted floor systems (87.8% of cases). Schulze Westerath et al. [43] reported that deep litter provides a soft and more comfortable surface for cattle, which was also confirmed by Wechsler [44]. It also offers better traction, which prevents lesions and swellings caused by slipping [45]. Housing on deep litter is recommended as a preventative measure of limb damage in heavy breeds [46]. It is, however, necessary to maintain the corresponding animal hygiene as part of the prevention of respiratory diseases, as has been stated above.

Chronic findings in limbs predominated among all classes of cattle, which confirms that musculoskeletal problems result from the conditions on the farm. The highest frequency of chronic findings in the limbs was recorded in cows, which may be the result of high production in dairy cows. Furthermore, Nash et al. [47] confirmed that limb lesions are a serious problem from the viewpoint of welfare on dairy farms.

Calves were another group affected by chronic changes in the limbs (4.16%). Disease of the limbs is a frequent reason for culling animals. Cerchiaro et al. [13] found damage to the musculoskeletal system to be the reason for culling of fattening bulls in 42% of cases. This figure was not corroborated in our study where bulls were the class in which pathological findings in the limbs were detected least frequently. Furthermore, the relatively low frequency of chronic findings in the limbs and their falling trend in cows, calves and heifers can be considered positive. This finding is favorable from the welfare point of view and reflects an increasing level of care for limbs on intensive cattle farms.

It is essential to farmers that animals are not only in good health but also in adequate body condition. The overall changes determined in our study were, first and foremost, emaciation, stunted growth and abscesses. Cachexia and stunted growth are reasons for the culling of animals. Ceccarelli et al. [12] recorded cachexia in 12.08% of slaughtered animals. Our results showed that emaciation was detected most often in calves (17.04%). It was recorded only at low frequencies in other classes. The reasons for emaciation are diarrheal and respiratory diseases which are not treated early enough or not at all. Calves are emaciated and their growth is stunted. Keeping such animals in the herd is economically disadvantageous for farmers. However, at the slaughterhouse, pronounced emaciation in calves is generally a reason for condemnation of the entire carcass [32]. Cachectic animals suffer from systemic diseases that manifest themselves during veterinary inspection in sensory abnormalities, icterus and septicemia. Farmers are aware of the implications and would rather discard such affected calves than keep them in the herd. Stunted growth is not detected very often at slaughterhouses, and this was confirmed by the results of our study, in which stunted growth was recorded in 3.49% of calves. In contrast, it was found at only a minimal level in other classes of cattle in comparison with calves (in less than 0.1%). Emaciation and stunted growth are closely associated with one another and point to the level of health of reared calves which is unfavorable over the long term from the viewpoint of respiratory and gastrointestinal infections and leads to these characteristic overall changes.

Abscesses, which arise as the result of the accumulation of pus in tissues largely due to bacterial infection, must also be stated among overall changes. Abscesses were recorded in only small numbers in our study. They were detected most often in cows (1.85%), and least often in heifers (0.70%). Different conclusions have been reached by foreign authors. According to Ceccarelli et al. [12], abscesses were a frequent finding, particularly in the liver (23.2% of cases) although their occurrence is variable and ranges between 10% and 20% of cases [48]. Amachawadi and Nagaraja [48] found abscesses to be a problem not only in beef cattle but also in dairy cows, which is due primarily to high-energy rations, rumen acidosis and excessive abundance of Gram-negative bacteria. Furthermore, Dupuy et al. [15] also considered abscesses to be a frequent finding, not merely in the liver, but also in other organs (lungs, kidneys etc.). According to Nagaraja and Lechtenberg [49], the prevalence of liver abscesses is linked to feeding practices. It is well known that concentrated feed increases the risk of liver abscesses compared to feed rich in fiber. Liver abscesses mainly affect feedlot cattle [50]. Most bulls slaughtered in the Czech Republic were animals aged between 12 and 24 months kept in extensive farming systems. The results showed that access to pasture and sufficient fiber reduces the risk of abscesses and liver findings, which were the least common in bulls compared to other classes. Changes in the liver were also mentioned by the European Commission [32], which described the formation of abscesses in calves. Biss et al. [51] monitored such findings in calves in New Zealand, where they recorded only a low incidence of liver abscesses. Although the previously published results indicate that abscesses, particularly in the liver, are a common finding at slaughterhouses, our results do not support this and ranks abscesses among isolated findings in all classes of cattle. The results indicate an improvement in the level of cattle welfare in relation to feed management.

The health of cattle can also be directly or indirectly affected by parasites. The developmental stages of some parasites and the pathological changes caused by them can be detected in infected animals during slaughterhouse veterinary inspection. However, reports on parasitic infection detected at slaughterhouses come mainly from non-EU countries [52,53]. The presence of parasitic findings may be related to the effectiveness of deworming programs on farms [54]. In our study, parasitic findings were detected only at low frequencies which is a positive finding. However, the low frequency of parasitic findings may result from the limited sensitivity of macroscopic veterinary inspections at slaughterhouses [55]. Slaughterhouse inspections can reveal flukes, tapeworms or gastrointestinal helminthes [54]. Our study confirmed that of all the organs monitored, liver and pancreas in heifers were most affected by parasitic changes. Before slaughter, heifers are often kept on pastures where they can become infected with parasites [56].

A very specific category of findings is tuberculoid changes, which may be related to the occurrence of bovine tuberculosis. Czech Republic was officially declared free of bovine tuberculosis in cattle on 31 March 2004 (Commission Decision No. 2004/320/EC) [57]. Diagnosis of bovine tuberculosis is currently carried out on farms by a tuberculin test. In suspected cases, samples are taken after slaughter for examination in the laboratory. During the monitored period (2010–2019), tuberculoid changes were recorded in 8 cows and 2 bulls. However, bovine tuberculosis was not confirmed by laboratory methods (histology, direct microscopy of homogenized tissue and culture) in the National Reference Laboratory for Bovine Tuberculosis.

Prospering and healthy animals are the precondition to successful cattle farming, the desired efficiency and, thereby, economic profit for farmers. A responsible approach to the treatment and prevention of health problems taken by farmers is critical. Not only are initiatives essential, but also practical implementation of adequate living conditions. The level of animal health and welfare is a reflection of the responsible work of every farmer.

## 5. Conclusions

The results of the study show differences in the spectrum and frequency of pathological findings between calves, heifers, cows and bulls. Detected findings also point to the differing health issues and differing state of health in the individual classes of cattle, which demand differing approaches to the treatment and prevention of the most frequent diseases. In cows, heifers and bulls, the most frequent chronic findings were made in the liver and pancreas, the lungs and the urinary tract. According to these pathological findings, it was confirmed that within adult cattle, cows were the most affected class because of great intensity of farming. Particularly dairy cows are at risk to a substantial metabolic burden. In contrast, the bulls had very good levels of the health. The smallest number of chronic and acute findings in the lungs was seen in bulls which confirms the fact that housing on pastures is highly beneficial. In calves, the most frequent findings were in the lungs, unclassified changes and overall changes, which point to the respiratory and diarrheal syndrome. These diseases can lead to loss of condition and emaciation which were detected in calves. Both chronic and acute findings in the lungs were recorded most often in calves at levels higher than those detected in adult cattle. The health problems of calves increase the costs of treatment and cause losses of animals as a result of culling. In addition, they present economic losses due to condemnations at the slaughterhouses. The spectrum of characteristic findings made in cows, heifers, bulls and calves is also a reflection of the level of welfare.

## Figures and Tables

**Table 1:** Number of pathological findings according to their localization in individual classes of cattle.

	Cows (*n* = 1,136,754)		Heifers (*n* = 257,912)		Bulls (*n* = 1,015,541)		Calves (*n* = 104,459)	
Finding	Number	%	Number	%	Number	%	Number	%
liver and pancreas	524,348	46.13 ^a^	38,136	14.79 ^c^	82,440	8.12 ^d^	17,880	17.12 ^b^
S and FS	91,642	8.06 ^a^	5141	1.99 ^b^	14,016	1.38 ^c^	1311	1.26 ^d^
intestines	31,740	2.79 ^a^	2419	0.94 ^c^	6880	0.68 ^d^	2406	2.30 ^b^
lungs	411,842	36.23 ^b^	45,407	17.61 ^c^	135,489	13.34 ^d^	46,894	44.89 ^a^
heart	104,919	9.23 ^b^	6719	2.61 ^c^	13,982	1.38 ^d^	10,524	10.07 ^a^
spleen	205,526	18.08 ^a^	20,144	7.81 ^b^	50,925	5.01 ^d^	6889	6.59 ^c^
reproductive organs	150,504	13.24 ^a^	684	0.27 ^b^	412	0.04 ^c^	5	0.00 ^d^
PAG and CD	1865	0.16 ^a^	319	0.12 ^b^	2	0.00 ^d^	4	0.00 ^c^
urinary tract	463,365	40.76 ^a^	41,905	16.25 ^c^	110,432	10.87 ^d^	17,774	17.02 ^b^
CNS	21	0.00 ^a^	12	0.00 ^b^	4	0.00 ^c^	4	0.00 ^a,b^
skin	214	0.02 ^c^	192	0.07 ^b^	64	0.01 ^d^	109	0.10 ^a^
head	3087	0.27 ^b^	444	0.17 ^c^	4680	0.46 ^a^	86	0.08 ^d^
trunk	42,507	3.74 ^a^	3579	1.39 ^c^	4934	0.49 ^d^	3331	3.19 ^b^
limbs	130,594	11.49 ^a^	12,513	4.85 ^c^	26,368	2.60 ^d^	7218	6.91 ^b^
overall changes	49,725	4.37 ^b^	4718	1.83 ^c^	9453	0.93 ^d^	22,509	21.55 ^a^
other changes	62,012	5.46 ^b^	4358	1.69 ^c^	6225	0.61 ^d^	6539	6.26 ^a^
unclassified changes	307,575	27.06 ^a^	19,591	7.60 ^c^	20,610	2.03 ^d^	25,515	24.43 ^b^

^a,b,c,d^ percentages in the same row with different superscripts differ (*p* < 0.05); S and FS = stomach and forestomach; PAG = pathological gestation; CD = congenital defects; CNS = central nervous system; n = number of slaughtered animals.

**Table 2:** Comparison of the occurrence of pathological findings in the livers of cows, heifers, bulls and calves slaughtered in slaughterhouses.

	Cows (*n* = 1,136,754)		Heifers (*n* = 257,912)		Bulls (*n* = 1,015,541)		Calves (*n* = 104,459)	
Findings	Number	%	Number	%	Number	%	Number	%
acute	59,195	5.21 ^a,w^	3488	1.35 ^b,x^	4343	0.43 ^c,x^	5379	5.15 ^a,w^
chronic	439,464	38.66 ^a,v^	30,474	11.82 ^b,v^	72,256	7.12 ^c,v^	12,242	11.72 ^b,v^
icterus	17,259	1.52 ^a,x^	299	0.12 ^b,y^	147	0.01 ^c,y^	120	0.11 ^b,x^
parasitic	8430	0.74 ^b,y^	3875	1.50 ^a,w^	5694	0.56 ^c,w^	139	0.13 ^d,x^

*n* = number of slaughtered animals. ^a,b,c,d^ percentages in the same row with different superscripts differ (*p* ˂ 0.01), ^v,w,x,y^ percentages in the same column with different superscripts differ (*p* < 0.01).

**Table 3:** Comparison of the incidence of pathological lung findings in cows, heifers, bulls and calves slaughtered in slaughterhouses.

	Cows (*n* = 1,136,754)		Heifers (*n* = 257,912)		Bulls (*n* = 1,015,541)		Calves (*n* = 104,459)	
Findings	Number	%	Number	%	Number	%	Number	%
acute	71,379	6.28 ^b,w^	3874	1.50 ^c,w^	10,690	1.05 ^d,w^	9635	9.22 ^a,w^
chronic	340,156	29.92 ^b,v^	41,503	16.09 ^c,v^	124,632	12.27 ^d,v^	37,219	35.63 ^a,v^
parasitic	307	0.03 ^b,x^	30	0.01 ^c,x^	167	0.02 ^c,x^	40	0.04 ^a,x^

*n* = number of slaughtered animals. ^a,b,c,d^ percentages in the same row with different superscripts differ (*p* ˂ 0.05), ^v,w,x^ percentages in the same column with different superscripts differ (*p* < 0.01).

**Table 4:** Comparison of the occurrence of pathological findings in the kidneys in cows, heifers, bulls and calves slaughtered in slaughterhouses.

	Cows (*n* = 1,136,754)		Heifers (*n* = 257,912)		Bulls (*n* = 1,015,541)		Calves (*n* = 104,459)	
Findings	Number	%	Number	%	Number	%	Number	%
acute	45,185	3.97 ^a,w^	2868	1.11 ^c,w^	4470	0.44 ^d,w^	3588	3.43 ^b,w^
chronic	417,875	36.76 ^a,v^	39,001	15.12 ^b,v^	105,841	10.42 ^d,v^	14,182	13.58 ^c,v^
parasitic	305	0.03 ^a,x^	36	0.01 ^b,x^	121	0.01 ^b,x^	4	0.00 ^c,x^

*n* = number of slaughtered animals. ^a,b,c,d^ percentages in the same row with different superscripts differ (*p* < 0.05), ^v,w,x^ percentages in the same column with different superscripts differ (*p* < 0.01).

**Table 5:** Comparison of the occurrence of pathological findings in the limbs in cows, heifers, bulls and calves slaughtered in slaughterhouses.

	Cows (*n* = 1,136,754)		Heifers (*n* = 257,912)		Bulls (*n* = 1,015,541)		Calves (*n* = 104,459)	
Findings	Number	%	Number	%	Number	%	Number	%
acute	35,257	3.10 ^a,w^	3601	1.40 ^c,w^	6860	0.68 ^d,w^	2486	2.38 ^b,w^
chronic	81,621	7.18 ^a,v^	7464	2.89 ^c,v^	17,239	1.70 ^d,v^	4341	4.16 ^b,v^
traumatic	13,716	1.21 ^a,x^	1448	0.56 ^b,x^	2269	0.22 ^d,x^	391	0.37 ^c,x^

*n* = number of slaughtered animals. ^a,b,c,d^ percentages in the same row with different superscripts differ (*p* < 0.01), ^v,w,x^ percentages in the same column with different superscripts differ (*p* < 0.01).

**Table 6:** Comparison of the incidence of overall changes in cows, heifers, bulls and calves slaughtered in slaughterhouses.

	Cows (*n* = 1,136,754)		Heifers (*n* = 257,912)		Bulls (*n* = 1,015,541)		Calves (*n* = 104,459)	
Finding	Number	%	Number	%	Number	%	Number	%
abscesses	21,000	1.85 ^a,w^	1800	0.70 ^c,w^	7309	0.72 ^c,v^	1032	0.99 ^b,x^
emaciation	28,035	2.47 ^b,v^	2627	1.02 ^c,v^	1773	0.17 ^d,w^	17,800	17.04 ^a,v^
stunted growth	41	0.00 ^d,x^	235	0.09 ^b,x^	249	0.02 ^c,x^	3647	3.49 ^a,w^

*n* = number of slaughtered animals. ^a,b,c,d^ percentages in the same row with different superscripts differ (*p* < 0.01), ^v,w,x^ percentages in the same column with different superscripts differ (*p* < 0.01).

## Data Availability

Data for analysis was obtained from the information system of the State Veterinary Administration of the Czech Republic. The datasets generated and analysed during the current study are available from the corresponding author on reasonable request.
